# The Cytostatic Action of Extracellular NAD in Tumour-bearing Mice

**DOI:** 10.1038/bjc.1972.40

**Published:** 1972-08

**Authors:** Siegmar Nolde, Helmuth Hilz

## Abstract

Repeated injections of NAD led to a dose-dependent inhibition of cell proliferation in tumour-bearing mice (Ehrlich ascites carcinoma and a murine mastocytoma). NAD proved clearly superior to other adenine nucleotides, including 3′,5′-cyclic AMP. Experiments with differently labelled NAD and studies on HeLa cultures showed that NAD is relatively slowly degraded by extracellular enzymes to AMP and adenosine, which probably represents the true cytostatic agent.

The superiority of NAD *in vivo* to other adenine nucleotides and to adenosine itself can be explained by the rate-limiting hydrolysis of NAD to AMP with a sustained production of cytostatic concentrations of adenosine. This may represent a new kind of “poisoning” by a potentially cytostatic compound brought about by the action of extracellular enzymes.


*Br. J. Cancer* (1972) **26**, 299.

### THE CYTOSTATIC ACTION OF EXTRACELLULAR NAD IN TUMOUR-BEARING MICE

SIEGMAR NOLDE AND HELMUTH HILZ

*Institut für Physiologische Chemie, Universität Hamburg, Germany*

Received for publication February 1972

**Summary.—Repeated injections of NAD led to a dose-dependent inhibition of cell proliferation in tumour-bearing mice (Ehrlich ascites carcinoma and a murine mastocytoma). NAD proved clearly superior to other adenine nucleotides, including 3′,5′-cyclic AMP. Experiments with differently labelled NAD and studies on HeLa cultures showed that NAD is relatively slowly degraded by extracellular enzymes to AMP and adenosine, which probably represents the true cytostatic agent.**

**The superiority of NAD *in vivo* to other adenine nucleotides and to adenosine itself can be explained by the rate-limiting hydrolysis of NAD to AMP with a sustained production of cytostatic concentrations of adenosine. This may represent a new kind of "poisoning" by a potentially cytostatic compound brought about by the action of extracellular enzymes.**

It has been shown by several groups that 3′,5′-cyclic AMP (cAMP) and other cyclic nucleotides are inhibitors of cell proliferation in various malignant cell lines *in vitro* and *in vivo* (Ryan and Heidrick, 1968; Gericke and Chandra, 1969; Heidrick and Ryan, 1970; Chandra, Gericke and Wacker, 1971). The physiological significance of these results is not clear, especially since we could demonstrate in HeLa cultures that exogenous NAD and other adenine derivatives were also potent inhibitors of cell proliferation (Nolde and Hilz, 1972). Even at $3 \times 10^{-5}$ mol/l concentrations NAD acted as a cytostatic, being somewhat superior to cAMP and other adenine nucleotides. NAD did not permeate the HeLa cells but was degraded extracellularly to AMP, adenosine and other derivatives. The pyridine-containing split products were not inhibitory. All the evidence pointed to adenosine as the true cytostatic agent. In cell culture experiments, adenosine was indeed the most potent cytostatic compound, followed by NAD and the other adenine derivatives. When we tried NAD as an inhibitor of tumour cell proliferation in mice, NAD proved to be clearly superior to adenosine and to adenine nucleotides. A cytostatic action of NAD has also been shown by Matsuyama, Maekawa and Nagayo (1961). The results presented here indicate a new kind of "poisoning" of a compound brought about by the action of extracellular enzymes.

### MATERIALS AND METHODS

The animals used were adult (25–30 g) female mice (Balb c). They were maintained in plastic cages on conventional laboratory feeds. The experimental tumours used were Ehrlich ascites carcinoma and a murine mastocytoma kindly supplied by Dr Karzel (Pharmakologisches Institut, Universität Bonn). Male mice bearing 6–7 day-old tumours served as donors. For the experiments, the original ascites fluid was diluted 1 : 10 with isotonic saline and groups of 10 mice were injected intraperitoneally with 0·1 ml of the dilution ($8{\cdot}5 \times 10^5 \pm 0{\cdot}14 \times 10^5$ cells). Treatment was started the day after inoculation, with 10 animals in each group. Each animal received 200 $\mu$l of isotonic saline—50 mM acetate buffer pH = 6·0, containing the freshly neutralized compound; the controls received buffered saline alone. Injections were repeated every second day; in some cases, injections were given daily. On termination of the experiment, the ascites of the whole group was pooled and analysed for cell number (Coulter counter), cell volume (haematocrit) and other parameters (see Fig. 1 and Tables).

Nucleotides were purchased from E. Merck, Darmstadt, and from Boehringer, Mannheim. $^3$H-adenine-labelled and $^3$H-nicotinamide-labelled NAD were synthesized as described previously (Nolde and Hilz, 1972).

FIG. 1.—Uptake of radioactive split product of differently labelled NAD by EAC cells. 0·1 ml ascites fluid containing $10^7$ cells was diluted with 0·9 ml modified Joklik medium (F-13, Grand Island, Biol. Co) and incubated at 37° C. 2·1 × $10^5$ cpm of $^3$H-adenine-labelled NAD or 2·0 × $10^5$ cpm of $^3$H-nicotinamide-labelled NAD were added, together with cold NAD, to give a final concentration of 1 × $10^{-4}$ mol/l; $10^{-4}$ mol/l nicotinamide was also present. Uptake of split products by the cells was determined as described previously (Nolde and Hilz, 1972).

RESULTS AND DISCUSSION

When mice bearing Ehrlich ascites carcinoma (EAC) cells were treated with repeated intraperitoneal injections of neutralized NAD solutions, a dose-dependent inhibition of tumour growth was observed (Table I). NAD proved to be superior to AMP and to adenosine in EAC cells (Table II). Nicotinamide (NAm), which is active as a cytostatic at very high concentrations (Matsuyama *et al.*, 1961), showed insignificant effects at these concentrations (Table II). In mastocytoma cells, NAD was somewhat less effective but always surpassed the action of adenosine or AMP. A representative experiment is presented in Table III.

TABLE I.—*Dose-dependent Inhibition of EAC Proliferation by NAD*

| Injections | Cells/mouse | % |
|---|---|---|
| Saline | $1·09 \times 10^8$ | 100 |
| NAD/10 μmol | $0·55 \times 10^8$ | 50 |
| NAD/40 μmol | $0·28 \times 10^8$ | 26 |

Intraperitoneal injections were given on the first, third and fifth day after transplantation (9·4 × $10^5$ cells). Cells were harvested at the seventh day, and counted as described in the text.

TABLE II.—*Inhibition of EAC Proliferation by Adenine Derivatives*

| | Relative cell proliferation | |
|---|---|---|
| Injections | % | *n* |
| Saline | 100 | 6 |
| NAD | 18±5 | 6 |
| NAm | 86 | 1 |
| AMP | 80±11 | 2 |
| ATP | 70 | 1 |
| Adenosine | 54±15 | 3 |

Mean and SEM values of the control groups (10 animals) were 1·86 ± 0·41 × $10^8$ cells per mouse (= 100%). Relative values for the experimental groups were based on the corresponding controls.

*n* = number of experiments. Daily injections with 0·02 ml of 0·2 mol/l neutralized solutions starting on the second day after inoculation (about 9 × $10^5$ cells) of animals (10 in each group). Cells were harvested at the sixth day.

TABLE III.—*Inhibition of Mastocytoma Proliferation in Mice by Adenine Derivatives*

| Injections | Cells/mouse | % |
|---|---|---|
| Saline | $3\cdot34\times10^8$ | 100 |
| NAD | $1\cdot96\times10^8$ | 59 |
| AMP | $2\cdot90\times10^8$ | 87 |
| Adenosine | $2\cdot47\times10^8$ | 74 |

Daily intraperitoneal injections of 40 μmol of the compounds in question were given from the first to the fifth day after transplantation ($1\cdot1\times10^6$ cells), with termination at the sixth day.

With the concentrations used, cell proliferation cannot be suppressed completely. Prolonged treatment kept values well below the controls, but the increase in weight due to an increase in ascites continued though at a reduced rate. NAD *per se* did not significantly alter the weight of normal animals (not shown).

The mechanism of inhibition must be different from the action of alkylating agents. While these lead to the formation of giant cells at concentrations producing partial inhibition of cell proliferation (Cohen and Studzinski, 1967; Schläger, Oldekop and Hilz, 1970), NAD (and other adenine derivatives) caused only slight increases in cell volume (Table IV). The treated cells also showed an elevated rate of $^3$H-thymidine incorporation into DNA (Table IV) in spite of the retarded cell multiplication, as has also been observed in HeLa cultures (Table VI).

The damage to EAC cells exposed to repeated NAD injections is indicated by an increased level of lactate dehydrogenase (LDH) in the ascitic serum as well as in the cells (Table IV).

NAD does not permeate the cells as an intact compound. When differently labelled NAD samples of the same specific radioactivity were exposed to EAC cells, the uptake of label was strikingly different (Fig. 1). This points to an extracellular degradation of NAD with a different uptake of the split products by the cells. An extensive extracellular hydrolysis of NAD also occurred in HeLa cultures.

The following arguments—obtained mainly by experiments with HeLa cultures—point to adenosine as the true cytostatic agent: NAD is degraded by the cell-free culture medium (containing calf serum) to AMP, adenosine and other split products (Table V). In HeLa cultures, all adenine derivatives so far tested proved to be cytostatic, adenosine at mmolar concentration being the most effective (Table VI). Other (non-adenine) nucleotides like NMN, IMP, GMP, UMP, FMN were not, or were substantially less, effective (not shown). All adenine derivatives, including adenosine, led to an increased incorporation of $^3$H-thymidine into DNA, when cultures were pretreated for 24 hours at mmolar (or lower) concentrations (Table VI). All cells detached from the glass surface are non-viable. Gentle centrifugation of the medium revealed broken cells only, which on re-explantation in fresh medium never proliferated, *i.e.* cell count of monolayers = total (viable) cell count. All adenine derivatives including (exogenous) NAD and adenosine produced a decrease in intracellular NAD levels (not shown). NAD, when applied in a DMSO solution to Harding–Passey melanoma in mice, again led to a significant retardation of tumour growth (Sloty *et al.*, 1971). At mmolar concentrations, adenosine was the most effective compound in HeLa cultures, while $3\times10^{-4}$ mol/l concentrations were considerably less effective (Table VI). In contrast, raising NAD levels above $3\times10^{-4}$ mol/l increased only slightly the cytostatic effect (or moderately—depending on the batch of serum used for the medium). This difference may be explained by differences in metabolism: adenosine is rapidly taken up by the cells and degraded or converted to purine nucleotides. To guarantee an effective level of the nucleoside during the 24-hour incubation period, a high concentration had to be applied. NAD on the other hand persisted much longer (Table V). The rate-limiting step for the degradation of NAD is the hydrolytic cleavage to AMP by a phosphodiesterase present in

TABLE IV.—*Alteration of Various Parameters in EAC Cells Treated* in vivo *with NAD*

| Parameter | Control group | NAD group | Ratio (NAD/control) |
|---|---|---|---|
| Cell proliferation (cells/mouse) | $1\cdot09\times10^8$ | $0\cdot28\times10^8$ | 0·26 |
| Cell volume ($ml\times10^{-9}$) | 2·36 | 2·85 | 1·21 |
| Thymidine incorporation into DNA (cpm/μg DNA) | 284±2 | 417±16 | 1·47 |
| Lactate dehydrogenase (U/ml ascitic serum) | 2·18±0·05 | 2·84±0·04 | 1·30 |
| ($U/10^8$ cells) | 38·1±2·0 | 46·7±1·4 | 1·30 |

Cell volume was calculated from "haematocrit" reading and cell number. DNA was determined according to Burton (1968). $^3$H-thymidine labelling of DNA was carried out with $6\times10^6$ pooled cells resuspended in 2·50 ml of fresh ascites serum of the corresponding group supplemented with 1 μC of $^3$H-thymidine (specific radioactivity 25 mCi/mmol, The Radiochemical Centre, Amersham). After incubation for 10 min at 37° C, acid insoluble radioactivity was determined according to Bollum (1959). LDH activity was determined according to Bergmeyer *et al.* (1962).

TABLE V.—*Extracellular Degradation of* $^3$*H-NAD by HeLa S3 Monolayer Cultures and by (Serum-containing) Medium*

| Adenine derivative in medium | $^3$H-radioactivity of NAD degradation products (cpm/ml medium) 0 hours | 6 hours | 24 hours |
|---|---|---|---|
| A. in the presence of medium only | | | |
| NAD | 439100 | 391380 | 286820 |
| AMP+IMP | 4550 | 45510 | 102340 |
| Adenosine+adenine | 4000 | 4550 | 22760 |
| Inosine+hypoxanthine | 9100 | 13650 | 47730 |
| B. in the presence of medium+cells | | | |
| NAD | 437200 | 362750 | 249750 |
| AMP+IMP | 4560 | 12650 | 8080 |
| Adenosine+adenine | 4500 | 8440 | 10060 |
| Inosine+hypoxanthine | 6880 | 37960 | 132800 |

$1\times10^{-3}$mol/l $^3$H-NAD (adenine-labelled, $6\cdot9\times10^6$ cpm/15 ml F-13 medium (GIBCo) containing 5% calf serum) was incubated in the presence or absence of $5\cdot5\times10^6$ cells (monolayer). At the indicated times, 450 μl samples of the medium were deproteinized, and aliquots chromatographed as described (Nolde and Hilz, 1972). The radioactive spots were eluted with 0·1 N HCl and radioactivity was analysed by liquid scintillation counting.

the serum-containing medium. In ascites serum, NAD at mmolar concentrations was degraded to about 80% during 24 hours at 37° C. The limited degradation of NAD may explain the clear superiority of the dinucleotide in tumour-bearing animals; while adenosine was rapidly removed from the peritoneal cavity, NAD persisted long enough to act as a reservoir for a continued production of cytostatic levels of adenosine. Thus, extracellular NAD represents a new form of a "latent" cytostatic compound (analogous to cyclophosphamide) which is slowly "activated" by enzymes of the extracellular space.

We wish to thank Mrs C. Winkens for competent technical assistance. This work was supported by the Bundesministerium für Bildung und Wissenschaft.

TABLE VI.—*The Action of NAD and Other Adenine Derivatives on Cell Proliferation and Thymidine Incorporation into DNA in HeLa Cultures*

| Additions | | Relative cell proliferation (increase in cells/flask) | | Relative $^3$H-thymidine incorporation into DNA (cpm/cell) | |
|---|---|---|---|---|---|
| None . . . . . | | 100 | ($n=17$) | 100 | ($n=9$) |
| NAD | $3\times10^{-4}$ mol/l | 25±5 | ($n=11$) | 179±26 | ($n=2$) |
| | $1\times10^{-3}$ mol/l | 7±1 | ($n=17$) | 225±13 | ($n=9$) |
| AMP | $1\times10^{-3}$ mol/l | 12±8 | ($n=6$) | 203±4 | ($n=6$) |
| ADP | $3\times10^{-4}$ mol/l | 32±21 | ($n=2$) | 154 | ($n=1$) |
| ADPR | $3\times10^{-4}$ mol/l | 35±7 | ($n=3$) | 203 | ($n=1$) |
| FAD | $3\times10^{-4}$ mol/l | 30±4 | ($n=2$) | 172±3 | ($n=2$) |
| Adenine | $3\times10^{-4}$ mol/l | 92±14 | ($n=2$) | — | |
| Adenosine | $3\times10^{-4}$ mol/l | 64±11 | ($n=8$) | 159±13 | ($n=5$) |
| | $1\times10^{-3}$ mol/l | 13±9* | ($n=7$) | 119±8 | ($n=3$) |

* Negative values represent cytotoxic action with detachment of cells from the glass surface in addition to complete inhibition of proliferation.

Mean and SEM values from separate experiments (each in duplicate) with monolayer cultures (about $2\cdot5\times10^6$ cell/flask, 10 ml F-13 medium containing 5% calf serum). The neutralized compounds were added for 24 hours. Proliferation was determined as the increase in cells/flask in 24 hours. Control values were $2\cdot3\pm0\cdot1\times10^6$ cells/flask. $^3$H-thymidine incorporation into DNA was measured by exposing cultures between hours 23 and 24 to 1 $\mu$Ci of $^3$H-thymidine (specific radioactivity 15·6 Ci/mmol, The Radiochemical Centre, Amersham, England). Incubation was stopped by pouring off the medium and washing the cells twice with 10 ml of ice-cold isotonic saline. The cell pellet was homogenized in 0·50 ml of 0·5 N NaOH (0°), 100 $\mu$l-aliquots were put on to filter papers and extracted according to Bollum (1959). Control values were $7\cdot50\pm0\cdot66\times10^3$ cpm/$10^6$ cells.

### REFERENCES

BERGMEYER, H. U., BERNT, E. & HESS, B. (1962) *Methoden der enzymatischen Analyse.* Ed. H. U. Bergmeyer. Weinheim: Verlag Chemie. p. 736.

BOLLUM, F. J. (1959) Thermal Conversion of Non-priming Deoxyribonucleic Acid to Primer. *J. biol. Chem.*, **234**, 2733.

BURTON, K. (1968) *Methods in Enzymology.* Vol. XII, *Nucleic Acids*, Part B. Ed. L. Grossmann and K. Modave, New York: Academic Press. p. 163.

CHANDRA, P., GERICKE, D. & WACKER, A. (1971) Effect of Nucleoside-(3′, 5′)-Monophosphates on Tumor Growth and Immunological Response in Mice. *VIIth International Congress of Chemotherapy, Prague* 1971.

COHEN, L. S. & STUDZINSKI, G. P. (1967) Correlation between Cell Enlargement and Nucleic Acid and Protein Content of HeLa Cells in Unbalanced Growth Produced by Inhibitors of DNA Synthesis. *J. cell. comp. Physiol.*, **69**, 331.

GERICKE, D. & CHANDRA, P. (1969) Inhibition of Tumor Growth by Nucleoside Cyclic 3′, 5′-Monophosphates. *Hoppe-Seyler's Z. physiol. Chem.*, **350**, 1469.

HEIDRICK, M. L. & RYAN, W. L. (1970) Cyclic Nucleotides on Cell Growth *in vitro*. *Cancer Res.*, **30**, 376.

MATSUYAMA, M., MAEKAWA, A. & NAGAYO, T. (1961) Inhibiting Effect of Nicotinamide and Diphosphopyridine Nucleotide on the Methylcholanthrene Sarcoma in Rats. *Nature, Lond.*, **189**, 673.

NOLDE, S. & HILZ, H. (1972) Extracellular NAD as a Cytostatic Agent. *Hoppe-Seyler's Z. physiol. Chem.*, **353**, 505.

RYAN, W. L. & HEIDRICK, M. L. (1968) Inhibition of Cell Growth in vitro by Adenosine 3′, 5′-Monophosphate. *Science, N.Y.*, **162**, 1484.

SCHLAEGER, R., OLDEKOP, M. & HILZ, H. (1970) Complete Dissociation of HeLa Cell Growth from Cell Division by an Alkylating Agent. *Hoppe Seyler's Z. physiol. Chem.*, **351**, 239.

SLOTY, A., PAPE, E., RHODE, B. & HILZ, H. (1971) Unpublished experiments.


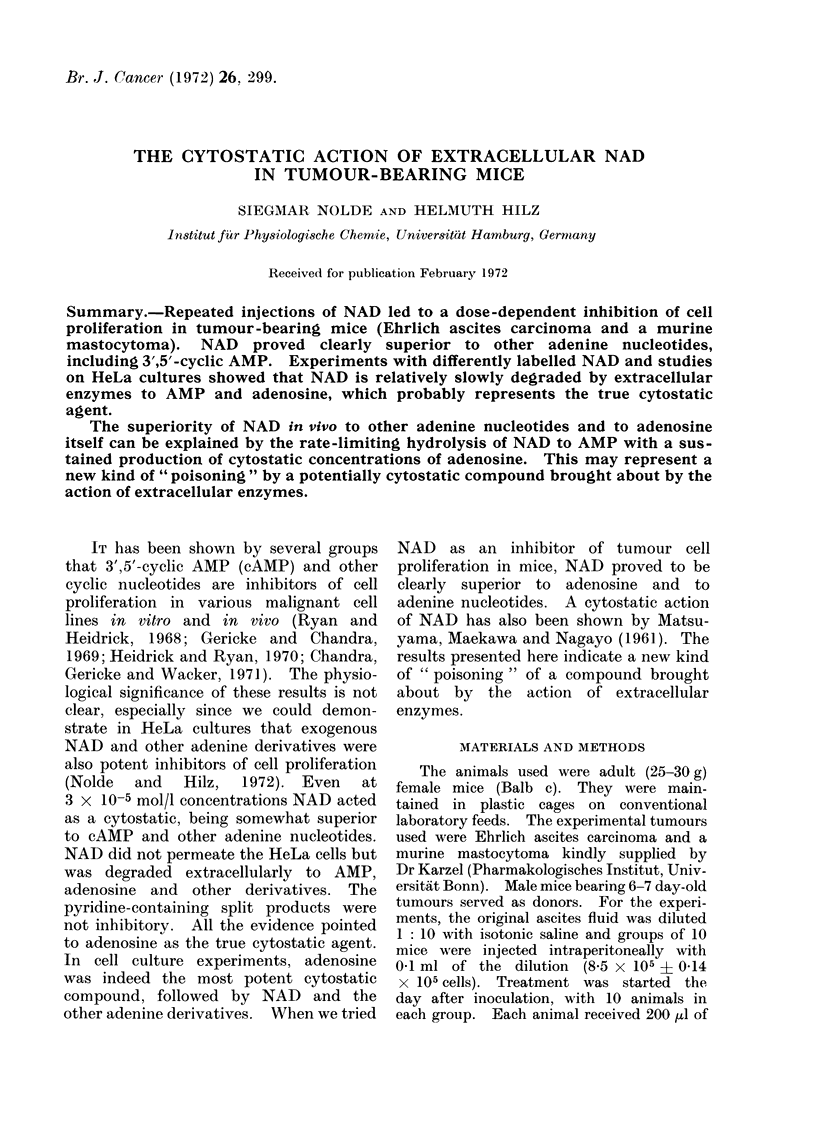

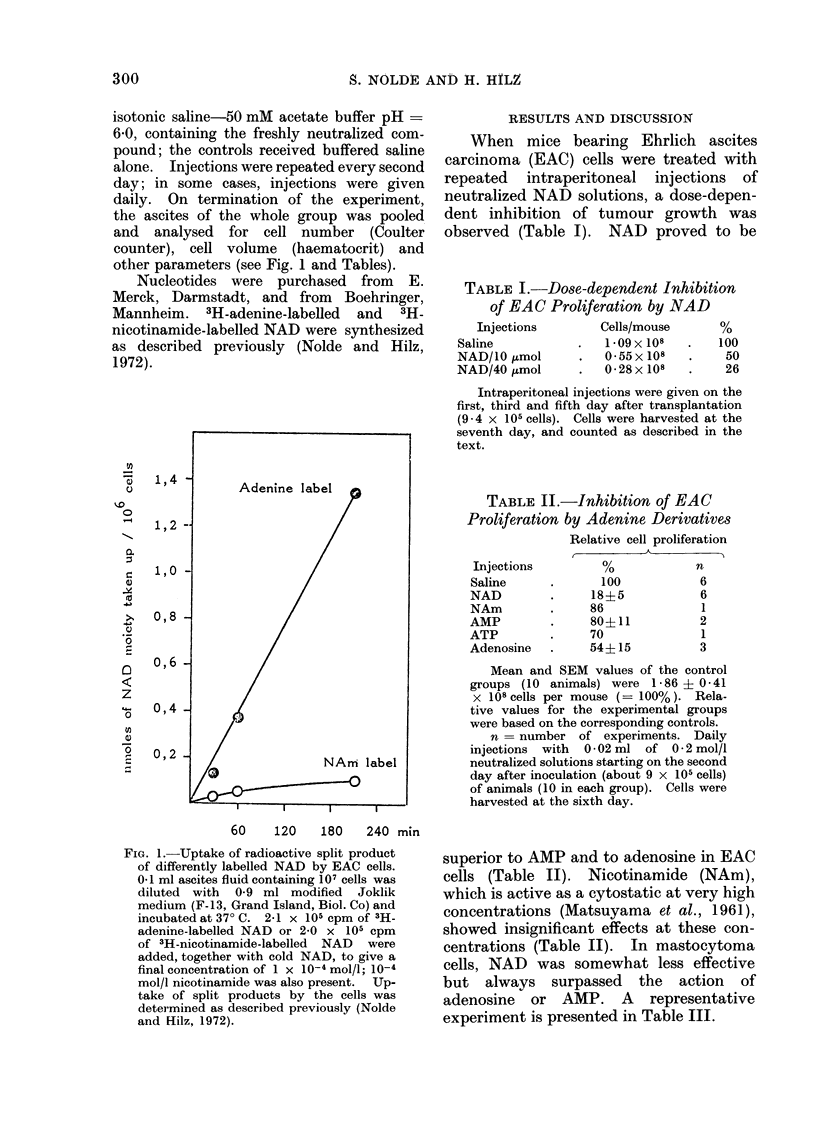

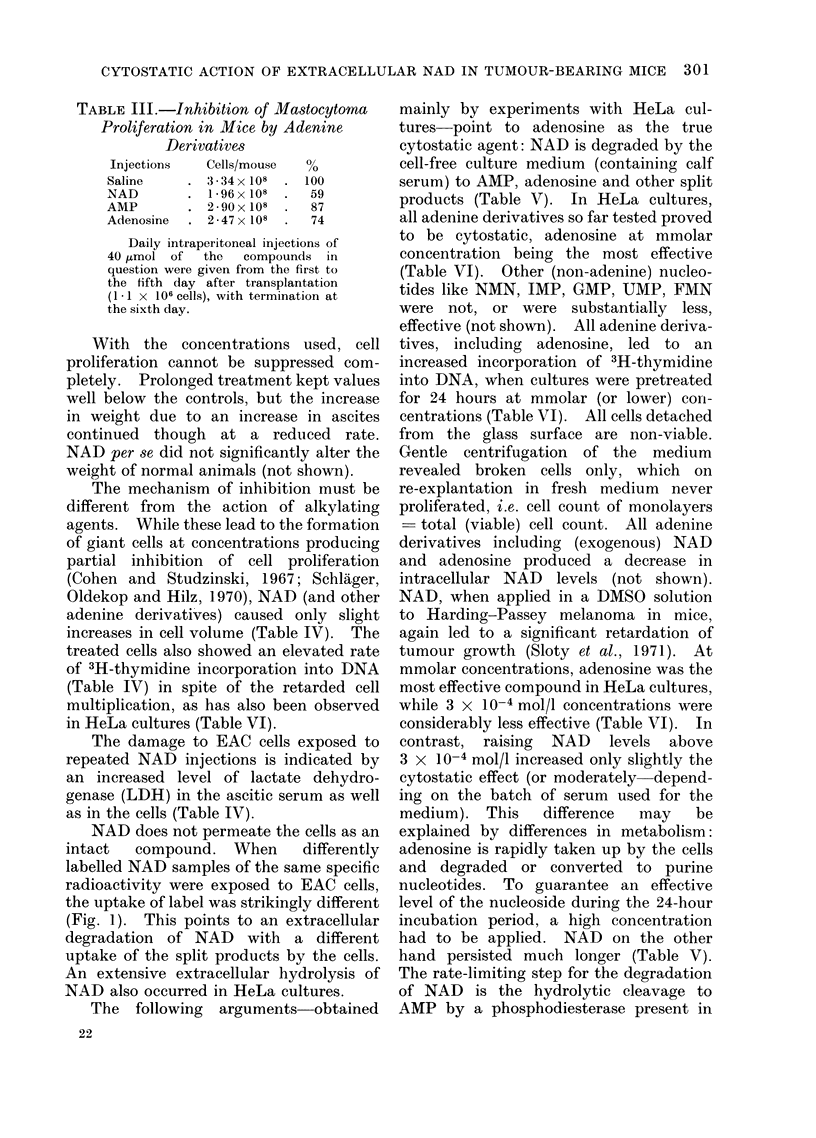

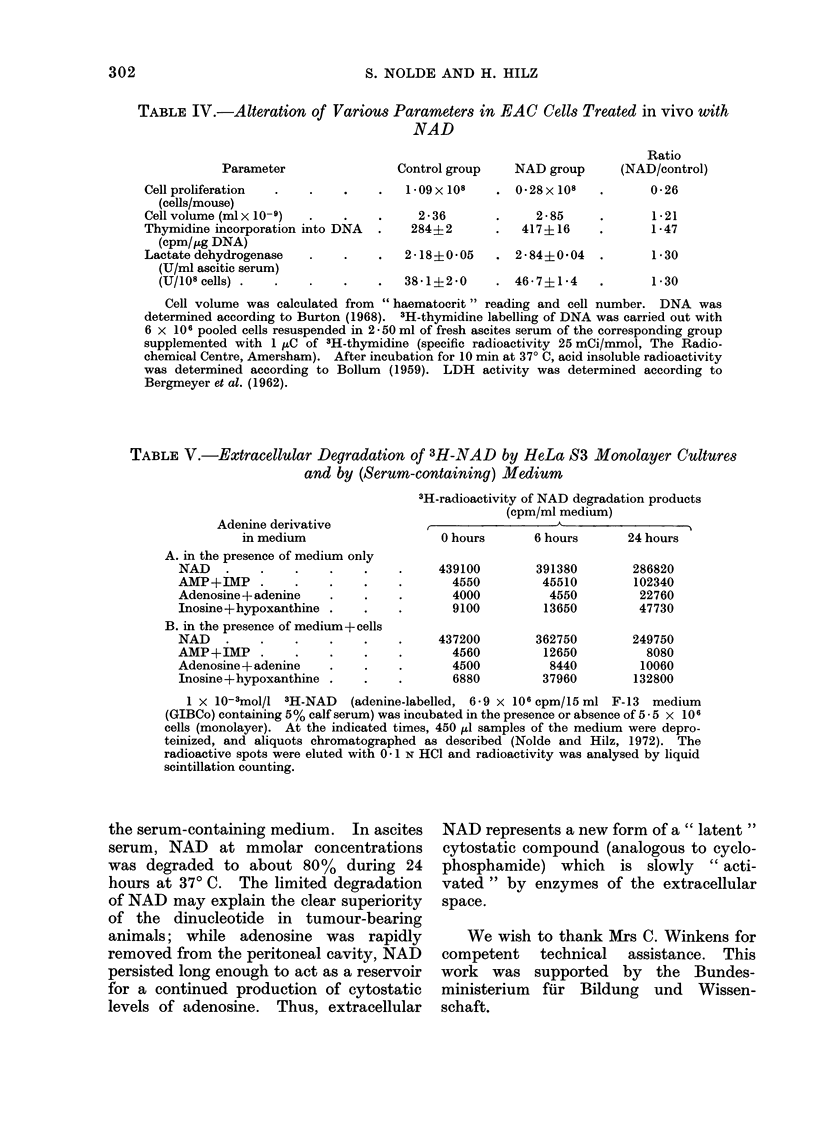

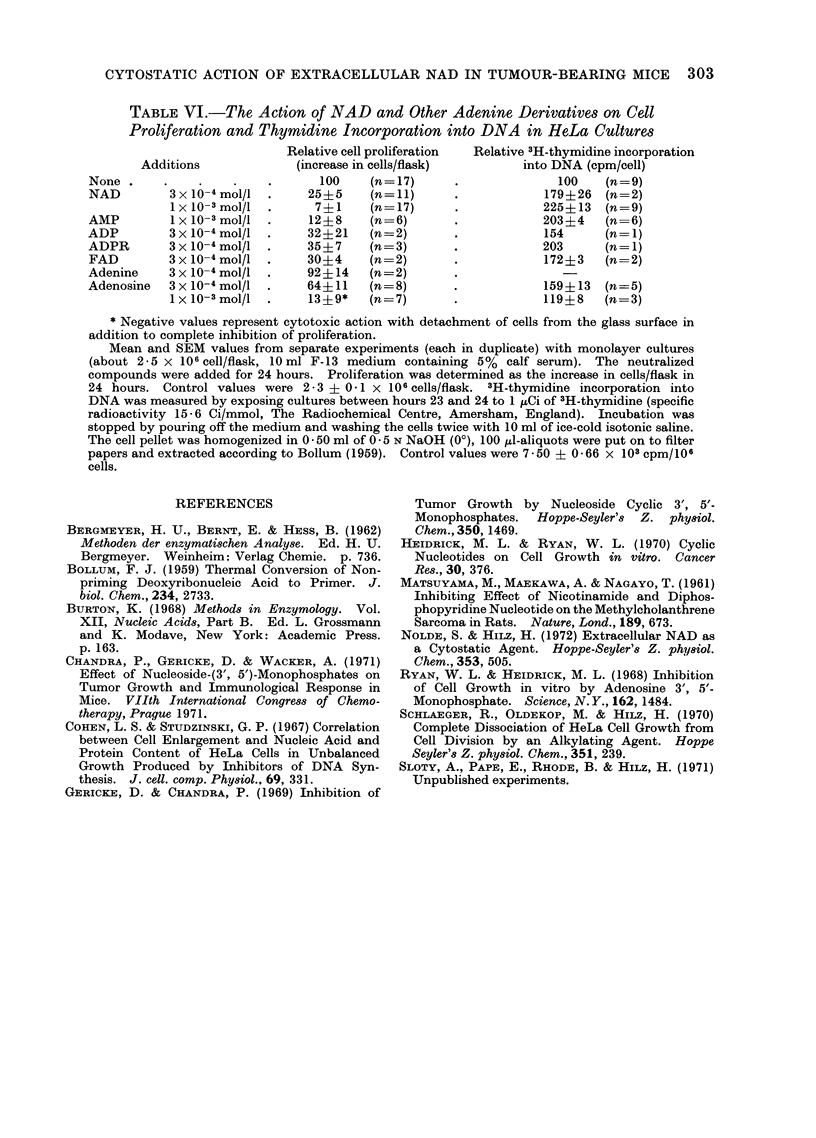


## References

[OCR_00545] BOLLUM F. J. (1959). Thermal conversion of nonpriming deoxyribonucleic acid to primer.. J Biol Chem.

[OCR_00563] Cohen L. S., Studzinski G. P. (1967). Correlation between cell enlargement and nucleic acid and protein content of HeLa cells in unbalanced growth produced by inhibitors of DNA synthesis.. J Cell Physiol.

[OCR_00570] Gericke D., Chandra P. (1969). Inhibition of tumor growth by nucleoside cyclic 3'-5'-monophosphates.. Hoppe Seylers Z Physiol Chem.

[OCR_00576] Heidrick M. L., Ryan W. L. (1970). Cyclic nucleotides on cell growth in vitro.. Cancer Res.

[OCR_00581] MATSUYAMA M., MAEKAWA A., NAGAYO T. (1961). Inhibiting effect of nicotinamide and diphosphopyridine nucleotide on the methylcholanthrene sarcoma in rats.. Nature.

[OCR_00587] Nolde S., Hilz H. (1972). Extracellular NAD as a cytostatic agent.. Hoppe Seylers Z Physiol Chem.

[OCR_00592] Ryan W. L., Heidrick M. L. (1968). Inhibition of cell growth in vitro by adenosine 3',5'-monophosphate.. Science.

[OCR_00597] Schlaeger R., Oldekop M. M., Hilz H. (1970). Complete dissociation of HeLa cell growth from cell division by an alkylating agent.. Hoppe Seylers Z Physiol Chem.

